# Classification of ADHD patients on the basis of independent ERP components using a machine learning system 

**DOI:** 10.1186/1753-4631-4-S1-S1

**Published:** 2010-06-03

**Authors:** Andreas Mueller, Gian Candrian, Juri D Kropotov, Valery A Ponomarev, Gian-Marco Baschera

**Affiliations:** 1 Brain and Trauma Foundation Grisons, Poststrasse 22, 7000 Chur, Switzerland; 2 Institute of the Human Brain of Russian Academy of Sciences, ul. Acad. Pavlova 9, 197376 St. Petersburg, Russian Federation

## Abstract

**Background:**

In the context of sensory and cognitive-processing deficits in ADHD patients, there is considerable evidence of altered event related potentials (ERP). Most of the studies, however, were done on ADHD children. Using the independent component analysis (ICA) method, ERPs can be decomposed into functionally different components. Using the classification method of support vector machine, this study investigated whether features of independent ERP components can be used for discrimination of ADHD adults from healthy subjects.

**Methods:**

Two groups of age- and sex-matched adults (74 ADHD, 74 controls) performed a visual two stimulus GO/NOGO task. ERP responses were decomposed into independent components by means of ICA. A feature selection algorithm defined a set of independent component features which was entered into a support vector machine.

**Results:**

The feature set consisted of five latency measures in specific time windows, which were collected from four different independent components. The independent components involved were a novelty component, a sensory related and two executive function related components. Using a 10-fold cross-validation approach, classification accuracy was 92%.

**Conclusions:**

This study was a first attempt to classify ADHD adults by means of support vector machine which indicates that classification by means of non-linear methods is feasible in the context of clinical groups. Further, independent ERP components have been shown to provide features that can be used for characterizing clinical populations.

## Background

Attention deficit hyperactivity disorder (ADHD) is a clinically heterogeneous neurobehavioral disorder that is associated with tremendous financial costs, stress to families, adverse academic and occupational outcomes. According to DSM-IV [1], the disorder is characterized by a varying amount of inattention, hyperactivity, and impulsivity symptoms. The ICD-10 [2], although using a different name, hyperkinetic disorder (HD), lists similar criteria for the disorder. The worldwide prevalence of the disorder is approximately 5% in children and adolescents [3]. ADHD symptoms decline over time [4], which may illustrate the developmental insensitivity of the DSM-IV [5,6]. However, more than one half of all ADHD children continue to display clinically significant symptoms after reaching adulthood [4]. With increasing age, the profile of the symptoms changes slightly, general difficulties involving the executive functions become more prominent than hyperactivity [7].

One of the most influential theoretical models of ADHD postulates that deficits in inhibitory control are the core of ADHD [8,9]. Brown [10,11] developed a comprehensive model to describe the complex cognitive functions impaired by the syndrome, wherein the impairments of the executive functions, the management system of the brain, are considered as being due to inherited problems in the chemistry of the system. The dynamic developmental behavioural theory [12] predicts that behaviour and symptoms in ADHD result from the interplay between individual predispositions and the surroundings, whereas hypofunctioning dopamine branches represent the main individual predispositions.

In this paper we examine the neurocognitive information processing system that can be defined by components of event related potentials (ERPs). These components represent sensory, memory and executive functions. The visual and auditory related sensory functions reflect early stages of information processing, and the executive functions are defined as the patient’s ability to plan, regulate and monitor his or her cognitive, emotional and motor skills in order to achieve certain goals. Executive control is of vital importance for all non-automated actions. This applies for acts which require the selection of an action, or for the inhibition of inappropriate impulses. Working memory functions maintain representations of stimuli and actions and compare the stage of the action to the expected response. The result of the current action can be anticipated and adaptive processes can be initiated. Our work is based on the concept of executive functions as described in more detail by Kropotov [13]. Basically, four functions can be distinguished: engagement operations, disengagement or inhibition operations, working memory, and monitoring operations.

ERPs allow the examination of the electrical representations of these underlying sensory and cognitive processes which occur in the brain. They contain a number of characteristic peaks and troughs which are associated with certain underlying processes. ERPs reflect phasic activity of cortical neurons. Activation, which originates from sensory stimuli, arrives to the primary cortical areas and starts a complex process of information flow through the cortico-subcortical networks. Activation of neurons in each cortical area is a result of inter-cortical interaction via feed-forward and feed-back pathways. In addition, the activity of cortical neurons is a result of interplay between excitatory and inhibitory cortical neurons. ERPs recorded from the scalp in response to stimuli appear to reflect these complex stages of information flow within the cortex.

ERPs have been investigated in a large number of studies in the context of sensory and cognitive-processing deficits in ADHD [14]. Differences have been reported both in auditory and in visual modality, in early as well as in late components, and using different paradigms. The GO/NOGO paradigm is primarily designed for the study of neurophysiological mechanisms of the brain’s executive functions [15]. Using this paradigm, Smith et al. [16] found group differences in the early stimulus processing components (P1, N1, P2). These abnormalities of children with ADHD were interpreted as showing problems with sensory registration and identification of stimuli. Broyd et al. [17], using an auditory GO/NOGO task as well, demonstrated enhanced N1 and P2 amplitudes, and reduced N2 amplitudes in ADHD children compared to controls. The results were interpreted as the ADHD group showing inhibitory deficiencies. Johnstone et al. [18], using a visual GO/NOGO task, found similar results. The ADHD group showed an increased parietal P2 amplitude, a reduced frontal N2 amplitude, and a more anterior P3 to NOGO stimuli, relative to GO stimuli, compared to controls. There are only a few ERP studies of adults with ADHD. Prox et al. [19] also used a visual GO/NOGO task and found that their adult ADHD group showed an increase in N1 and N2 amplitudes and a slight P3 reduction. They interpreted these results in terms of the patients compensating for their impairments by focusing their attention more strongly than controls. Fallgatter et al. [20], in adult ADHD subjects, reported reduced anteriorisation of the NOGO N2 and a reduced increase of P3 amplitudes in NOGO trials compared to controls. They interpreted these results in terms of prefrontal brain dysfunction related to response inhibition and/or cognitive control.

In this study, independent component analysis (ICA) was used to decompose ERPs into a set of independent components. ICA is a computational method that separates a set of mixed potentials measured at the scalp into a corresponding set of statistically independent source signals [21]. Using a modification of the visual two-stimulus GO/NOGO paradigm, Kropotov, Ponomarev [submitted] separated classical ERP waves into independent components which were associated with a specific functional meaning.

There have been several attempts to discriminate ADHD from control subjects on the basis of EEG (for a review see [22]) and ERP data [23-26], by using linear classification methods, with moderate classification accuracy. Smith et al. [26] concluded that ERP information could be of considerable diagnostic utility in children, but less in adolescents, and that it might be implemented in clinical practice as an additional diagnostic tool.

In recent years, new classification algorithms, such as neural networks or support vector machines (SVM), have emerged in the field of electroencephalography. When using such non-linear classifiers, nonlinear relationships in the feature data that are not obvious may be found. The support vector machine [27], a method originating from machine learning, has been used in the context of automated spike analysis [28], artefact detection and removal [29], EEG pattern recognition [30] and evoked potentials [31-34]. Support vector machines are learning systems that use pre-classified training data, and then apply the results to test data. The system uses a non-linear mapping kernel function to transform the data into a higher dimensional space, where the data is believed to be linearly separable. Classification is then performed by constructing a hyperplane that maximizes the separating margin between the closest samples of the two classes.

Löfhede [35] compared three different classification algorithms by applying them to the epochs of periodic burst-suppression EEG activity from six full-term newborn infants which had suffered from perinatal asphyxia. The SVM showed the best performance in detecting bursts, followed by artificial neural network (ANN) and Fisher’s linear discriminant (FLD). However, the slight advantage in performance was accompanied by higher computational complexity. Merzagora et al. [36] compared the performances of linear and non-linear classifiers on different sets of ERP features (P300 and N200 amplitudes) with regard to their ability to accurately characterize and discriminate these features as responses to target and non-target stimuli. In general, non-linear and non-parametric classifiers (quadratic classifier, multi-layer perceptron neural network, support vector machine) performed better than linear classifiers (Euclidean classifier, Mahalanobis discriminant, Fisher’s linear discriminant). The authors concluded that the methods investigated can provide an objective approach to detecting and diagnosing abnormalities and evaluating interventions for clinical populations.

This study aimed to examine the potential of independent ERP components as objective features for the classification of adult ADHD patients, using a non-linear support vector machine classifier.

## Methods

### Subjects

Two groups, each consisting of 34 female and 40 male subjects between the ages of 18 and 50 years, participated in the study. The mean age in the ADHD group was 34.1 years (SD 9.1). The mean age in the sex- and age-matched control group was 34.6 years (SD 9.2). Subjects were included in the ADHD group only if they had been diagnosed with ADHD prior to study participation by an independent psychiatrist. ADHD diagnosis was confirmed at study intake on the basis of the DSM-IV criteria for ADHD [1]. Due to the adult sample, the DSM-IV criteria were modified on one point according to Barkley et al. [7]: The DSM-IV primarily requires that an individual have at least 6 of 9 listed symptoms of inattention or 6 of 9 listed hyperactive/impulsive symptoms to qualify for the diagnosis of ADHD. In the present study, subjects were included in the ADHD group if they retrospectively recalled the presence of at least 4 frequently occurring inattention and/or hyperactivity/impulsivity symptoms in childhood (ages 5-12 years), and if at least 4 inattention and/or hyperactivity/impulsivity symptoms had been frequently present during the past 6 months before study participation.

The clinical assessment included a series of ADHD questionnaires, as well as a diagnostic interview (see procedure section). The determination of the presence of adulthood ADHD symptoms in the clinical interview resulted in 21 subjects being the inattentive, 4 subjects being the hyperactive/impulsive, and 49 subjects being the combined ADHD subtype. Again, the presence of 4 symptoms on the inattention and/or the hyperactive/impulsive lists were used as basis for the subtype classification. Except for symptoms of psychosis, comorbidities were no reason for subject exclusion. Subjects were unmedicated, or they had refrained from taking methylphenidate during 24 hours before testing. Subjects taking other psychotropics were not included in the study. Also, subjects which had suffered of a head injury with subsequent loss of consciousness, and subjects suffering from neurological or systemic medical diseases were excluded from the study. 66 ADHD subjects were right-handed, 8 ADHD subjects were left-handed.

The control group consisted of 74 healthy subjects recruited from the local community and matched for sex and age. Subjects were recruited by advertising the study in local media, companies and associations. Exclusion criteria corresponded to the criteria used for the ADHD group, i.e. subjects which had suffered of a head injury with subsequent loss of consciousness, and subjects suffering from neurological or systemic medical diseases were excluded from the study. Furthermore, control subjects had to score lower than the level of clinical significance on a symptom checklist [37]. No control subjects were receiving medication at time of testing. 66 control subjects were right-handed, 6 control subjects were left-handed, and 2 subjects were ambidextrous.

The study was approved by the local ethics committee and written informed consent was obtained from all participants after an explanation of the procedure.

### Procedure

Prior to the first session, ADHD subjects were asked to complete and return a series of questionnaires, including the Brief Symptom Inventory [37], a health history questionnaire [38], and the Current and Childhood Symptoms Scales [38]. The latter rating scales were, if available, also obtained from the subject’s parents (Childhood Symptoms Scale) and partners (Current Symptoms Scale). Then the subjects were tested in a first session which lasted approximately three hours. During this period, a comprehensive structured clinical interview [38] was carried out, comprising of an assessment of current and past ADHD symptoms, the history of problems at school, the past psychiatric history (including drug and medication use), as well as past and present comorbidities. Subsequently, EEG data was acquired. EEG data was first recorded while the subject was in an eyes-closed and in an eyes-open resting condition, lasting four minutes each. Then data was recorded while subjects were performing a visual continuous performance task (VCPT), which is the focus of this paper. The execution of the VCPT took approximately 22 minutes. In addition, subjects randomly performed either an auditory or an emotional continuous performance task. In a second session, a series of neuropsychological tasks was administered to the subjects. All of these tasks are not relevant to this paper.

The control group had a shortened procedure. Subjects were tested in a single session lasting approximately two and a half hours. During this period, a series of questionnaires (Brief Symptom Inventory [37], Health History questionnaire [38], Current Symptoms Scales [38]) were filled out and thereafter, EEG data was acquired. Subsequently, a working memory task, which is not relevant to this paper, was administered to the subjects.

EEG was recorded using a Mitsar 201 (Mitsar Ltd.), a PC-controlled 19-channel electroencephalographic system. The input signals referenced to the linked ears were filtered between 0.5 and 50 Hz and digitized at a sampling rate of 250 Hz. Impedance was kept below 5 kOhm for all electrodes. Electrodes were placed according to the International 10-20 system using a electrode cap with tin electrodes (Electro-cap International Inc.). Quantitative data was obtained using WinEEG software. Linked ears reference montage was changed to average reference montage prior to data processing. Eyeblink artefacts were corrected by zeroing the activation curves of individual ICA components corresponding to eyeblinks [39]. In addition, epochs of the filtered electroencephalogram with excessive amplitude (>100 μV) and/or excessive fast (>35 μV in 20 to 35 Hz band) and slow (>50 μV in 0 to 1 Hz band) frequency activities were automatically marked and excluded from further analysis. Finally, EEG was manually inspected to verify artefact removal.

### Behavioral task

**Figure 1:**
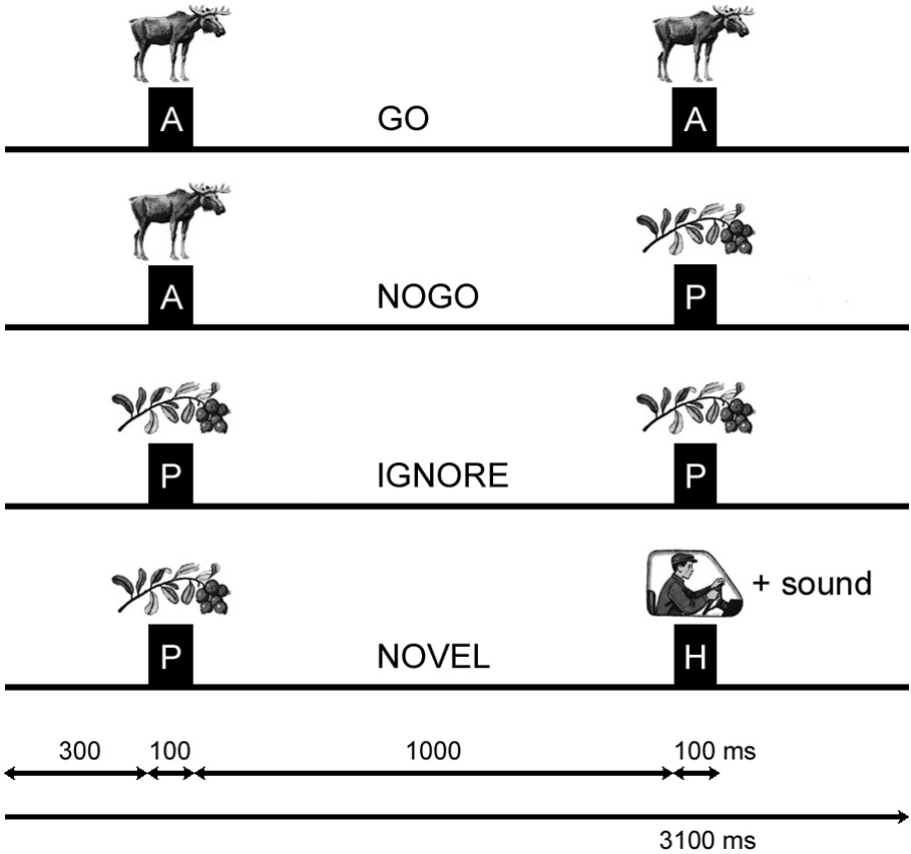
**Schematic representation of the two stimulus GO/NOGO task** Time dynamics of stimuli in four trial categories. Abbreviations: A, P, H are stimuli consisting of pictures of animals, plants and humans. GO trials require the subject to press a button. NOGO trials require suppression of a prepared action. IGNORE trials are stimuli pairs beginning with a picture of a plant, which require no preparation for action. NOVEL trials are stimuli pairs requiring no action, with the presentation of a novel sound together with the second human picture stimulus. Time intervals are depicted at the bottom.

The VCPT is a modification of the visual two-stimulus GO/NOGO paradigm. Three categories of visual stimuli were selected: 20 pictures of animals, 20 pictures of plants, and 20 pictures of humans (presented together with an artificial “novel” sound). The trials consisted of presentations of pairs of stimuli (see figure 1): animal-animal (GO trials), animal-plant (NOGO trials), plant-plant (IGNORE trials), and plant-human (NOVEL trials). The trials were grouped into four blocks. In each block a unique set of five animal stimuli, five plant stimuli and five human stimuli was selected. Each block consisted of a pseudo-random presentation of 100 stimuli pairs with equal probability for each trial category. The task was to press a button as fast as possible in response to all GO trials.

According to the task design, two preparatory sets were distinguished in the trials. In the “Continue set” a picture of an animal is presented as the first stimulus and the subject is supposed to prepare to respond; in the “Discontinue set” a picture of a plant is presented as the first stimulus and the subject does not need to prepare to respond.

During the task, subjects were seated in a comfortable chair, 1.5 m in front of a computer screen. The stimuli were presented on a 17 inch monitor using the Psytask (Mitsar Ltd.) software.

### Decomposition of collection of ERPs into independent components

The goal of independent component analysis (ICA) is to utilize the differences in scalp distribution between different generators of ERP activity to separate the corresponding activation time courses [40]. Components are constructed by optimizing the mutual independence of all activation time curves, leading to a natural and intuitive definition of an ERP component as a stable potential distribution which cannot be further decomposed into independently activated sources. The advantages of ICA for EEG analysis in comparison to other blind source separation approaches like principal component analysis (PCA) are discussed in detail elsewhere [41]. Briefly, PCA suggests that components should not be correlated and uses only second-order statistics (the data covariance matrix). PCA, looking for orthogonal axes ranked in terms of maximum variance, completely misses the data structure.

ICA uses statistics of all orders and pursues a more ambitious objective. In contrast to PCA, ICA can better separate a mixture of several nonorthogonal distributions that are independent.

In this study, ICA was performed on the full ERP scalp location x time series matrix.  Assumptions that underlie the application of ICA to individual ERPs are as follows: 1) summation of the electric currents induced by separate generators is linear at the scalp electrodes; 2) spatial distribution of the components’ generators remains fixed across time, 3) generators of spatially separated components are temporally independent from each other [40,41]. In addition to these assumptions we suggest that cortical locations are similar between individuals, so that it is viable to implement the ICA on an array of ERPs for a group of subjects.

There is at least one reason to use ERPs as input data instead of the usage of concatenated single trial data. It is known that the amplitude of ERPs is lower than the amplitude of EEG signals. Hence, independent component decomposition of concatenated single trial data will mainly separate independent components related to EEG sources. The averaging of single trials reduces the amplitude of spontaneous EEG activity and extracts signals which are evoked by the presentation of stimuli and by performing the task. Therefore the application of ICA to averaged ERPs allows us to focus on ERP related components ignoring spontaneous EEG sources.

The input data are the collection of individual ERPs arranged in a matrix *P* of 19 channels (rows) by *T* time points (columns). The ICA finds an ´unmixing´ matrix (*U*) that gives the matrix *S* of the sources (independent components) when multiplied by the original data matrix (*P*)

S = UP

where S and *P* are 19 x *T* matrices and *U* is 19 x 19 matrix. *S*(t) are maximally independent. Matrix *U* is found by means of the Infomax algorithm [40]. Infomax ICA exploits temporal independence of source signal waveforms to perform blind separation, by finding a square “unmixing” matrix by gradient ascent that maximizes the joint entropy of a nonlinearly transformed ensemble of zero-mean input vectors (see [42]).

According to linear algebra,

*P = U*^-1^*S*,

where *U*^-1^ is the inverse matrix of *U* (also called mixing matrix). Further,

*P* = ∑*P*_i_ = ∑*U*_i_^-1^*S*_i_,

where *U*_i_^-1^ is the i-th column of the mixing matrix *U*^-1^ and represents the topography of the i-th independent component; *S*_i_ is the row of the *S*-time course corresponding to the i-th independent component.

The i-th independent source *S*_i_ can be found as

*S*_i_ = *U*_zi_*P*,

where *U*_zi_ is matrix *U* in which all rows are zeroed except the i-th row.

According to linear algebra,

*P*_i_ = *U*^-1^* U*_zi_*P*,

where *U*^-1^* U*_zi_ is a filter (19 x 19 matrix) for extracting the i-th component from the matrix *P* of multi-channel potential timeseries. This filter was used for decomposing individual ERP waves into the independent components. 

The spatial filters used in this study have been constructed within the framework of another study by two of the authors [Kropotov, Ponomarev, submitted]. In that study, which used the same experimental task, the independent components were extracted from a collection of 297 individual ERPs of healthy subjects aged 18 to 45. The independent components were constructed in response to the second stimuli of the VCPT trials in a time interval after the presentation of the second stimulus. The ICA of the ERPs was made separately for Continue sets and Discontinue sets. All the components remained stable when ICA was applied to two randomly divided subgroups of the whole sample.

For the present study, we selected seven independent components from the component set extracted in Kropotov, Ponomarev [submitted]. These components were selected based on their power on the one hand, explaining 76% of the total power of grand average ERP signals, and on the basis of a solid background with regard to their functional meaning on the other hand. Three of the selected components were not dependent on the preparatory set, three of the components were specific for the continue set (GO and NOGO conditions), and one component was specific for the novelty condition. As to the set-independent and continue-set-specific components, only NOGO condition time courses were included in the feature extraction procedure.

### Locating the generators of the independent components

For the process of locating the generators of the independent ERP components, the sLORETA [43] imaging approach was used. The names of the components were derived from the localization area determined by sLORETA.

### Feature extraction

For the classification of subjects into ADHD subjects and controls we have to define features, which can be extracted from the independent ERP components. Each feature operates within a certain time window of a given independent component. It locates minimum or maximum values within the time window and returns either the extremal amplitude or latency. To reduce the bias in the selection of features based on expert knowledge in ERP analysis, we defined feature templates, which allow to automatically generate a set of features. A feature template consists of a start and end point in time, a time window size, and a time step size. Additionally, it contains the information regarding the type of extremal point (min/max), which has to be located, and also the information regarding the type of return value (amplitude/latency) the feature provides. Based on these feature templates, the set of possible features is extracted as follows: For each feature template we set the time window from the given start point to the start point plus time window size on the specified ERP component. Within this time window, the extremal point is located and the specified return value is computed. Subsequently, the time window is shifted by the time step size and the feature extraction procedure is repeated until the time window reaches the defined end point in time.

### Support vector machine (SVM)

**Figure 2:**
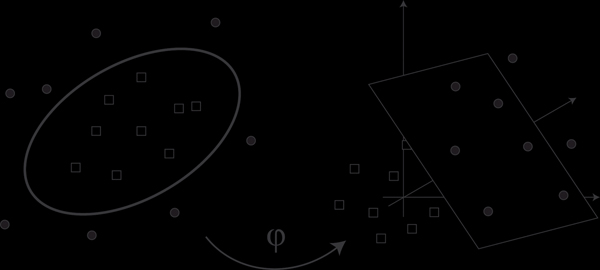
**Projection of input feature space into a higher dimensional space** The classification process of two samples (circles and squares) is complex in 2 dimensions (left) and simple in three or more dimensions (right). The elements close to the hyperplane are called support vectors.

The task given to a supervised learner is to provide the best possible separation of predefined groups in a multidimensional space after having seen a number of training examples. A common approach for such a classification is to explicitly model the boundaries between the classes as a separating hyperplane, as realized, for example, in the perceptron model [44]. Similarly, support vector machine (SVM) tries to identify the hyperplane with the largest margin to all data points. This positioning of the hyperplane is only dependent on the closest data points, which are called support vectors.  However, these approaches are restricted to linearly separable data. To overcome this restriction, the input feature space can be projected into a higher dimensional space. This allows for a linear separation of previously linearly inseparable data (see figure 2). The projection into a higher dimensional space and the corresponding identification of the optimally separating hyperplane is computationally intensive. Support vector machines reduce the computational cost by a kernel trick [45]. The scalar product in the higher dimensional space, required to fit the maximal-margin hyperplane, is replaced by a non-linear kernel function in the lower dimensional space. This allows for a computation of the separating hyperplane in the high dimensional space and provides a non-linear classifier in the low dimensional space, without the actual, computationally expensive transformation of the support vectors. The most popular of these kernel functions is the radial basis function, which has been employed for the SVM classification based on the ERP components in this publication. For comparison, classification performance was tested using a linear kernel as well. A more detailed description of support vector machines can be found in [46].

### Model selection

The presented support vector machine builds a classifier based on a given set of features. However, the number of extracted features for each subject based on different time windows, feature types and ICA components is very large and would result in an overfitting of the SVM classifier. To select an appropriate set of features we have to consider the predictive power of the resulting classification routine. A popular technique to assess how the results of a statistical analysis will generalize to an independent data set is cross-validation [47]. The main idea behind cross-validation is to split the data k-times for an estimation of the performance of each classifier: k-1 parts of the data are used for training each classifier and the remaining part is used to test the predictive power (in this work, a 10-fold validation was used). The optimal set of features and its corresponding classifier is determined by a forward selection scheme, where the best M features are iteratively selected from the set of N possible features. The time complexity of this model selection is O(N*M), i.e., the algorithm runs linearly in number of possible features and requested size of optimal feature set.

### Statistical analysis of behavioural and independent component data

Student’s t-test was used for assessing statistical significance of the group differences related to the psychometric test results, the behavioural performance, and to the independent components amplitudes. 

## Results

### Clinical and behavioural data

Mean data for the clinical scales are presented in table 1. The ADHD group scored significantly higher on the Current Symptoms Inattention subscale (*t*(146)=25.838, *p*<.001), the Current Symptoms Hyperactivity/Impulsivity subscale (*t*(146)=16.668, *p*<.001), the Current Symptoms Total ADHD scale (*t*(146)=26.940, *p*<.001), as well as regarding the BSI General Severity Index (*t*(146)=14.219, *p*<.001). As to the comorbidities in the ADHD group, the following concerns were reported most frequently: significant changes to sleep pattern (28.4%), prolonged periods of depression (25.7%), significant appetite changes (21.6%), excessive anxiety (13.5%), and excessive fears (13.5%).

**Table 1:** Mean *(sd)* psychometric test results for the ADHD and Control groups

	ADHD	Controls
Current DSM-IV inattentive symptoms	6.12 *(2.00)*	0.07 *(0.25)*
Current DSM-IV hyperactive/impulsive symptoms	4.51 *(2.27)*	0.08 *(0.28)*
Current DSM-IV total ADHD symptoms	10.64 *(3.33)*	0.15 *(0.39)*
Childhood DSM-IV inattentive symptoms	6.91 *(1.87)*	-
Childhood DSM-IV hyperactive/impulsive symptoms	4.04 *(2.69)*	-
Childhood DSM-IV total ADHD symptoms	10.95 *(3.76)*	-
BSI General Severity Index	1.30 *(0.67)*	0.17 *(0.14)*

Table 2 shows the behavioural performance of the participants in the VCPT. The ADHD group showed a significantly higher number of omission errors (*t*(146)=5.125, *p*<.001) and a significantly higher RT variance (*t*(146)=5.275, *p*<.001), compared to the control group. The groups did not differ in the other variables.

**Table 2:** Mean *(sd)* behavioural VCPT performance for the ADHD and Control groups

	ADHD	Controls	Total
Omission errors (GO)	4.36 *(4.93)*	1.23 *(1.71)*	2.78 *(3.99)*
RT GO	420.41 *(83.81)*	419.42 *(94.06)*	419.91 *(88.78)*
Standard error of mean RT	11.03 *(3.83)*	8.20 *(2.55)*	9.61 *(3.54)*
Commission errors (NOGO)	.64 *(1.02)*	.45 *(.80)*	.54 *(.91)*

### Independent ERP components

**Figure 3:**
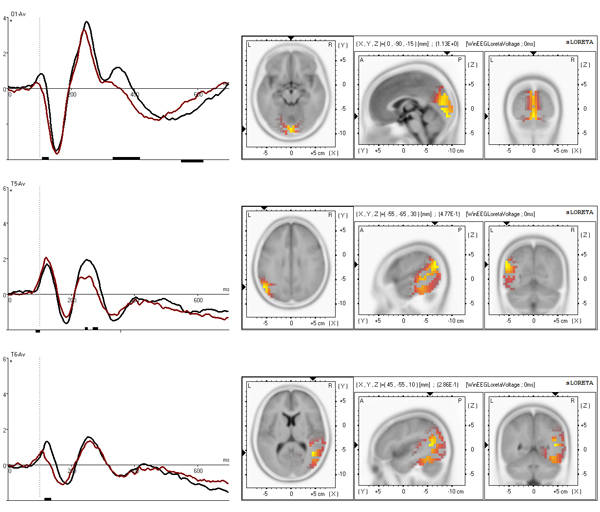
**Sensory related independent components** Top: BA 18 component at O1. Middle: BA 39 left component at T5. Bottom: BA 39 right component at T6. Time courses (left) are presented separately for control (black) and ADHD (red) group. x axis is time in ms, y axis is amplitude in μV. Results of t-statistics are presented below the curves with vertical bars corresponding to p<0.05. SLORETA imaging is presented on the right.

**Figure 4:**
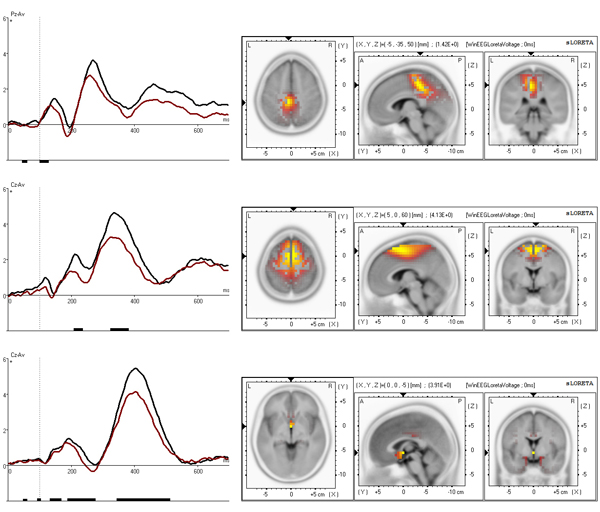
**Executive independent components** Top: BA 5 component at Pz. Middle: BA 6 component at Cz. Bottom: BA 25 component at Cz. Time courses (left) are presented separately for control (black) and ADHD (red) group. x axis is time in ms, y axis is amplitude in μV. Results of t-statistics are presented below the curves with vertical bars corresponding to p<0.05. SLORETA imaging is presented on the right.

**Figure 5:**
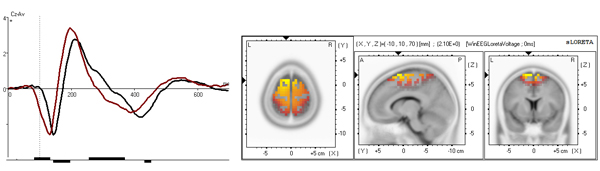
**Novelty related independent component** BA 6 novelty component at Cz. Time course (left) is presented separately for control (black) and ADHD (red) group. x axis is time in ms, y axis is amplitude in μV. Results of t-statistics are presented below the curve with vertical bars corresponding to p<0.05. SLORETA imaging is presented on the right.

The grand average time courses of the selected independent components, separated for the control group and the ADHD group, are presented in figures 3, 4 and 5, left. According to sLORETA, maximal current source densities for the three set-independent components are distributed over the Lingual Gyrus of the occipital lobe for the first component (BA 18), and over the left and right temporal-parietal junctions for the second (BA 39 left) and third (BA 39 right) components (figure 3, right). The three continue-set-specific components are located in the middle parietal cortex (BA 5), in the supplementary motor cortex (BA 6 NOGO), and in the anterior part of the cingulate cortex (BA 25). The NOGO time courses and the localization of these components are illustrated in figure 4. While the parietal component is larger for the GO condition, the premotor and cingulate components are larger for the NOGO condition [Kropotov, Ponomarev, submitted]. The novelty condition component (BA 6 novelty) is located in the premotor cortex (figure 5, right).

### Feature extraction

For each of the selected seven independent components, only the time course at one single site was entered in the feature extraction procedure. The site selection was based on the amplitude prominence of the respective components at the different 19 sites and can be found in figures 3, 4, 5. The final feature set was automatically generated using the following feature template settings: The start point of the component’s time course was 0 ms, the end point was set at 600 ms. Time window size was 100 ms (+/-25%, and +/-50%). Time step size was set at 4 ms. The feature set selected by the automated feature selection algorithm, composed of a combination of five features with best classification performance, without exception consisted of latency values of minimum amplitude. Four independent components were comprised in the feature set: the BA 6 novelty, BA 18, BA 25, and BA 5 components. The time windows were as follows: 112-160 ms for BA 6 novelty component, 292-364 ms for BA 18 component, 480-528 ms for BA 25 component, 488-588 ms and 440-540 ms for BA 5 component.

### Classification

Using the non-linear SVM with a 10-fold cross-validation approach, the classification accuracy of assigning ADHD subjects and controls to the corresponding groups was 92% (sensitivity 90%, specificity 94%). Classification performance using a linear SVM was 90%.

## Discussion

Because objective diagnostic access is not widely available, diagnoses in clinical practice often rely on the subjective observations and perceptions of the patients, and on the personal diagnostic criteria of the clinician. This study is intended to make a contribution to the improvement of objectivity in diagnosing ADHD. For that purpose, we used a modification of the visual two stimulus GO/NOGO task in order to obtain ERP responses in four task conditions. Using generic spatial filters built on the basis of a set of 297 healthy subjects, independent component analysis was applied to individual ERPs and combined with a non-linear classification method in the context of adult ADHD patients. We investigated whether features of independent ERP components can be used to accurately discriminate adults with ADHD and control subjects. In doing so, we decomposed ERP responses of the different task conditions into a set of seven independent components (ICs) and entered the resulting time courses into a non-linear support vector machine.

Classification performance was good, with a 10-fold validated accuracy of 92%. Using a linear kernel function, classification performance was 90%. The ICs that were entered into the feature selection procedure consisted of three task condition independent components, three continue-set-specific (GO and NOGO conditions) components, and one novelty condition specific component. Based on their specificity for the preparatory set, their time dynamics, and their localization, these ICs could be divided into sets of visual (BA 18, BA 39 left, BA 39 right), executive (BA 5, BA 6 NOGO, BA 25) and novelty (BA 6 novelty) components [Kropotov, Ponomarev, submitted]. The first of the task condition independent components was localized in the occipital lobe and resembles the visual N1 wave described in previous studies [48]. The other two condition-invariant ICs were localized over the left and right hemisphere temporal-parietal junctions and appear to correspond to the occipito-temporally distributed N170 waves described in numerous studies on ERP correlates of object processing [49]. The parietally distributed continue-set conditions specific IC matches the corresponding parameters of conventional P300 waves, which have been associated with context-updating and memory operations [50]. The other two continue-set-specific ICs, both larger for the NOGO condition, were distributed centrally. The first of these NOGO-condition-specific components was localized in the premotor cortex, a part of the cortex involved in motor inhibition [51], and thus may be associated with the inhibition of a prepared motor action in response to NOGO cues. The other NOGO-condition-specific component exhibits a strong negative peak at 270 ms when contrasted to GO cues. This negative deflection may be related to the N2 NOGO wave [52,53], which has been associated with conflict monitoring [54]. The novelty condition specific component has a central distribution and its positive deflection peaking at around 210 ms corresponds to the novelty component found in previous studies using conventional current density mapping [55] and ICA performed on single trial EEG epochs [56]. Goldstein et al. [57] hypothesized that the novelty P3 component could reflect the inhibition of a response engaged automatically with the detection of a deviant event, a model which is supported by the localization of the present novelty component in the premotor cortical area.

The automated feature selection algorithm determined a set of five features to belong to the optimal combination for classification. Looking at the feature set extracted by the automated procedure, three observations deserve attention.

First, all of the features consisted of extremal latency values in specific time windows. They corresponded to the N1 peak of the BA 6 novelty component and to late waves of the BA 18, BA 25 and BA 5 components. In contrast to deviations in amplitude, latency differences are rarely reported in the ADHD ERP literature. Keage et al. [58] reported shorter latencies of the P3a ERP component in children and adolescents with ADHD and interpreted this finding in terms of an ineffective evaluation of deviant stimuli resulting in an increased distractibility. This finding corresponds to the differences found in the present study regarding the BA 6 novelty component. However, due to the resemblance to the BA 6 NOGO component regarding topography and localization, we tend towards an interpretation in terms of the executive system inhibiting processes spuriously triggered by an early detection of deviance [57]. The uncommon predominance of latency features compared to amplitude features in discriminating ADHD and control subjects may be explained in terms of the wide age range of the subjects in the present study, in that there is a considerable change of ERP amplitudes with age [Kropotov, Müller, Candrian, Ponomarev, in preparation].

Secondly, independent components associated with different functional systems of the brain were included in the set of extracted features. Besides the executive functions, which are supposed to be the core area in the context of ADHD difficulties [8-11] and which were covered by the BA 25 and BA 5 components, sensory (BA 18 component) and novelty related (BA 6 novelty component) functions seem to play a certain role, too, with regard to the cognitive impairments in ADHD. This finding is corroborated by a number of studies showing deviations in ADHD subjects with regard to early sensory related [14,16] and novelty related [58-60] ERP components.

Thirdly, the extracted feature set comprised two very similar features of the BA 5 component. This finding shows that the feature selection procedure has to be refined in future studies. The ameliorations are to be concerning the selection of an optimal number of features highly correlated with the class, yet uncorrelated with each other.

Classification performance based on the 10-fold cross-validation procedure was remarkable and supports the use of non-linear classifiers in characterizing patient groups on the basis of ERP features. However, the results have to be further validated by using an independent test sample. A study according to that topic is planned. Classification performance using a linear SVM was marginally minor. Future studies will have to show which kind of approach is more useful for the characterization of ADHD patients and the interpretation of the respective electrophysiological deviations.

In contrast to studies using conventional ERPs, to our knowledge, so far there have been no attempts to discriminate ADHD from control subjects on the basis of independent ERP components gained by means of ICA. The present study shows that this endeavour is practicable with good success. Yet, this study does not allow for a comparison between the two options with regard to classification performance. Future studies are needed to investigate this question.

One main limitation of the present study is the use of spatial filters constructed within a different sample of healthy subjects for decomposing individual ERPs into independent components. This decision was taken with regard to the robustness of the independent components in view of about 300 subjects in the mentioned sample. Although the results of the present study show that independent ERP components can be used for classifying ADHD patients, the use of generic spatial filters limits the significance of the results with regard to the electrophysiological characteristics of ADHD patients.

This study is a first attempt to classify ADHD patients by means of support vector machine and independent ERP components, and despite the successful result, independent component decomposition and feature extraction procedures have to be refined in future studies. However, this promising approach can easily be applied to other clinical problems.

## Conclusions

This study was a first attempt to classify ADHD patients by means of support vector machine. The encouraging result of the study indicates that classification by means of non-linear methods is feasible in the context of clinical groups. Thereby, independent ERP components have been demonstrated the ability to provide features that can be used in characterizing clinical populations.

## Competing interests

GC, GMB report no potential conflicts of interest.

AM, JDK, VAP are members of the board of HBImed company. HBImed provides accesses to a referential database.

## Authors' contributions

AM, GC, JDK carried out the study design, GC, AM headed the recording, test and interview procedures with the patients. GC, AM, JDK, GMB drafted the manuscript. GMB programmed the support vector machine for ERP data. VAP programmed ICA ERP decomposing. All authors read and approved the final manuscript.
